# Musculoskeletal Health in the 21^st^ Century

**DOI:** 10.1186/1471-2474-16-S1-I1

**Published:** 2015-12-01

**Authors:** Ali Mobasheri

**Affiliations:** 1 School of Veterinary Medicine and Science, School of Veterinary Medicine, Faculty of Health and Medical Sciences, Manor Park Campus, University of Surrey, Guildford, GU2 7AL, UK; 2 Arthritis Research UK Centre for Sport, Exercise and Osteoarthritis, Arthritis Research UK Pain Centre, Medical Research Council and Arthritis Research UK Centre for Musculoskeletal Ageing Research, University of Nottingham, Queen's Medical Centre, Nottingham, NG7 2UH, UK; 3 Center of Excellence in Genomic Medicine Research (CEGMR), King Fahd Medical Research Center (KFMRC), Faculty of Applied Medical Sciences, King AbdulAziz University, Jeddah, 21589, Kingdom of Saudi Arabia

## 

In July 2015 a multidisciplinary two-day workshop entitled “Musculoskeletal Health in the 21st Century” was organised by the authors at the University of Surrey. The aim of the workshop was to bring together some of the major stakeholders including clinicians, basic scientists and funding bodies to focus on current challenges in musculoskeletal health and discuss current strategies for intervention and disease prevention. Workshop participants discussed and debated the effects of physical activity, body condition, diet and vitamins on the musculoskeletal system, focusing specifically on the synovial joint. The workshop also included sessions on joint health, arthritis prevention through physical activity (including biomechanics of musculoskeletal tissues), effects of diet and nutrition, understanding the underlying physiology and pathophysiology of cartilage and bone, prognostic biomarkers and new insights from genetic diseases of the musculoskeletal system. This Special Supplement of BMC Musculoskeletal Disorders includes a general review article summarising some of the current research in musculoskeletal health and includes abstracts presented at the July 2015 workshop in Surrey.

